# The *atroviolacea* Gene Encodes an R3-MYB Protein Repressing Anthocyanin Synthesis in Tomato Plants

**DOI:** 10.3389/fpls.2018.00830

**Published:** 2018-06-19

**Authors:** Sara Colanero, Pierdomenico Perata, Silvia Gonzali

**Affiliations:** PlantLab, Institute of Life Sciences, Scuola Superiore Sant’Anna, Pisa, Italy

**Keywords:** anthocyanin, *atv*, MBW complex, R3-MYB, repression of anthocyanin production, *Solanum lycopersicum*, tomato

## Abstract

The anthocyanin biosynthetic pathway is well characterized in plants. However, in tomato (*Solanum lycopersicum* L.) an exhaustive knowledge of its regulation is still lacking. Tomato mutants showing higher levels of anthocyanins in fruits or vegetative tissues, such as *Anthocyanin fruit (Aft*) or *atroviolacea* (*atv*), have been extensively exploited in the attempt to clarify the process. Nevertheless, only candidate genes have been proposed as responsible for such phenotypes. The recessive *atv* mutation likely represents an allelic variant of a gene introgressed in tomato from wild *Solanum* species. We performed genome sequencing of *atv/atv* plants followed by candidate gene analysis, and identified a mutated gene encoding an R3-MYB protein. When overexpressed, this protein abolished anthocyanin production in tomato seedlings and plants, by silencing key regulators and biosynthetic genes of the pathway. The functional analysis of the protein clearly showed that it can negatively interfere with the activation of the anthocyanin biosynthetic pathway mediated by the endogenous MYB-bHLH-WDR (MBW) complexes. In particular, this R3-MYB protein can directly bind the bHLH factors which are part of the MBW complexes, therefore acting as a competitive inhibitor. The R3-MYB protein here described is therefore involved in a feedback mechanism that dampens the production of anthocyanins once activated by endogenous or exogenous stimuli. The *atv* mutation causes the production of a truncated version of the R3-MYB factor that cannot retain the full potential to inhibit the MBW complexes, thus leading to a constitutively higher production of anthocyanins.

## Introduction

Anthocyanins are water soluble pigments mainly synthesized in epidermal and sub-epidermal cells of shoots, roots, flowers, and fruits. They represent the glycosylated forms of the anthocyanidins, secondary metabolites belonging to the class of flavonoids and synthesized through the phenylpropanoid pathway (Winkel-Shirley, 2001). Anthocyanins are antioxidant compounds able to protect leaves from high light intensity and other stressful conditions (Gould, 2004). In flowers and fruits their attractive colors, spanning from red to blue, facilitate pollination and seed dispersion (Glover and Martin, 2012).

Experimental evidence has been accumulating in the years supporting an important role of anthocyanins as well as other classes of plant antioxidants as health-promoting compounds. Anthocyanins can prevent cardiovascular diseases, improve visual and brain functions, control body fat accumulation and diabetes, and have anti-atherosclerotic, anti-carcinogenic, and antiviral effects which might be related to specific anti-inflammatory properties (Tsuda, 2012). Recently, cyanidin, one of the most common anthocyanidin, has been shown to alleviate inflammation *in vivo* thanks to its specific capacity to inhibit signaling by the proinflammatory cytokine interleukin-17A (Liu et al., 2017). In this sense anthocyanins can be considered as nutraceuticals and the edible vegetables and fruits containing them are functional foods.

Anthocyanins are produced through a branch of the flavonoid biosynthetic pathway whose enzymatic steps are well characterized (Winkel-Shirley, 2001). Developmental and environmental factors can both induce anthocyanin production with a well-defined tissue and cellular patterning (Albert et al., 2014b). Complex regulatory mechanisms respond to the different inducing signals activating and repressing the biosynthetic pathway at transcriptional and post-translational levels (Albert et al., 2014a; Xu et al., 2015). A central role in the expression of the biosynthetic genes is carried out by specific transcription factors (TFs). Some of them are R2R3-MYB TFs which operate as transcriptional activators in combination with basic-helix-loop-helix (bHLH) and WD-repeat (WDR) proteins in the so-called MBW complex (Baudry et al., 2004; Koes et al., 2005; Ramsay and Glover, 2005; Xu et al., 2015). An increasing number of regulatory proteins has been characterized in recent years. They also include negative regulators, either R2R3-MYB or R3-MYB proteins, which can disrupt the activity of the MBW complex by directly sequestering specific factors participating to it and indirectly suppressing the expression of genes encoding components of the same complex (Albert et al., 2014a; Wang and Chen, 2014). Petunia PhMYB27 and PhMYBx represent clear examples of R2R3-MYB and R3-MYB repressors, respectively, recently characterized (Albert et al., 2014a).

Contrary to other *Solanaceae*, such as pepper or eggplant, tomato (*Solanum lycopersicum* L.) fruits cannot accumulate anthocyanidins due to the incomplete activation of the flavonoid biosynthetic pathway (Verhoeyen et al., 2002). However, the synthesis of anthocyanins can take place in tomato fruits when TFs from snapdragon are ectopically expressed via genetic engineering (Butelli et al., 2008). The ability to accumulate anthocyanins in fruit peel was also introgressed in *S. lycopersicum* from wild *Solanum* relatives by spontaneous hybridizations (Gonzali et al., 2009), leading to the production of “blue” or “purple” tomatoes by conventional breeding. Some of these new tomato genotypes originated from a cross between the *Aft* or the *Aubergine* (*Abg*) accession with the *atv* line (Mes et al., 2008; Gonzali et al., 2009; Scott and Myers, 2012).

Whereas *Aft* and *Abg* likely represent two different alleles of a dominant gene encoding an R2R3-MYB TF activating the anthocyanin biosynthesis (Georgiev, 1972; Rick et al., 1994; Jones et al., 2003; Schreiber et al., 2012), the genetic identity of the *atv* gene has remained unknown for a long time. Originally described as a spontaneous hybrid between *S. lycopersicum* and *S. pimpinellifolium* (Rick et al., 1968), *atv* mutant is more likely derived from a Galapagos Islands accession of *Solanum cheesmaniae* (Kendrick et al., 1997; Jones et al., 2003). The *atv* mutation confers an increased response to light in terms of hypocotyl growth inhibition and anthocyanin synthesis, especially under red or far-red light: this led to the hypothesis that it might be involved in the phytochrome signaling (Kendrick et al., 1997). *atv*/*atv* plants show intense anthocyanin pigmentation in leaf veins, stems and green fruits also when grown in cool conditions (Rick et al., 1968). The intermediate intensity of coloration shown by the *ATV*/*atv* heterozygous plants did not allow to undoubtedly map the locus in a classical segregation analysis. However, linkage tests with some genetic markers supported the association with chromosome 7 (Rick et al., 1968; Clayberg, 1972). Very recently, an R3-MYB encoding gene located on the long arm of chromosome 7 has been identified as linked to the mutation by a fine-mapping approach and was therefore proposed as a candidate gene underlying the *atv* mutation (Cao et al., 2017).

A transcript profiling analysis carried out in *atv*/*atv* green fruits revealed a sustained transcription of the EBGs of the phenylpropanoid pathway leading to the synthesis of both flavonol and anthocyanin precursors (Povero et al., 2011). However, when *atv* is combined with the *Aft* mutation, which mainly affects the expression of the LBGs, most of the LBGs show an even higher upregulation, indicating that *atv* gene acts synergistically with *Aft* on LBG transcription, by inducing itself the same genes or exerting a transcriptional de-repression (Povero et al., 2011). This leads to the strong anthocyanin pigmentation shown by *Aft*/*Aft atv*/*atv* fruits, especially when exposed to high light and/or cool conditions (Mes et al., 2008; Povero et al., 2011; Scott and Myers, 2012; Cao et al., 2017).

In this paper we experimentally demonstrate that the *atv* phenotype is genetically associated with the mutation of the gene encoding the R3-MYB protein recently identified on chromosome 7 and named *SlMYB-ATV* (Cao et al., 2017). We show that the protein acts as a repressor of anthocyanin synthesis, in a feedback inhibition mechanism that disrupts the activity of the MBW complexes initiating the production of the pigments.

## Materials and Methods

### Plant Materials and Growth Conditions

Tomato (*S. lycopersicum* L.) variety AC (accession LA2838A, Tomato Genetic Resource Center, TGRC, University of California, United States), cv. MT, *atv*/*atv* line in MT background (Sestari et al., 2014), in VF36 background (accession LA0797, TGRC) and in AC background (accession LA3736, TGRC), as well as double mutant *Aft*/*Aft atv*/*atv* (Povero et al., 2011) were used. Tomato seeds were germinated in rock wool plugs (Grodan, Roermond, Netherlands) soaked in a nutritive solution (Kiferle et al., 2013). 2-week-old seedlings were transplanted in plastic pots containing a mixture of 70:30 soil (Hawita Flor, Vechta, Germany)/expanded clay (Vialca Srl, Pistoia, Italy), and placed in a growth chamber with 12 h light photoperiod, 100 μmol (protoplast isolation)/300 μmol (anthocyanin extraction and RNA isolation) photons m^-2^ s^-1^ irradiation intensity, 24°C/21°C day/night temperature, 50% relative humidity. Apical leaves were sampled, frozen in liquid nitrogen and stored at -80°C until use.

*Arabidopsis thaliana* (L.) Heynh. ecotype Columbia 0 (*Col-0*) was used. Seeds were germinated in plastic pots containing soil (Hawita Flor) and plants were cultivated under 8 h light photoperiod, 80 μmol photons m^-2^ s^-1^ irradiation intensity, 23°C/20°C day/night temperature, 50% relative humidity. Seeds collected from plants subjected to floral dip were surface-sterilized with diluted bleach and germinated in 1% agar plates containing 0.5X MS medium (Murashige and Skoog, 1962) and kanamycin. Kanamycin-resistant seedlings were then transferred to soil and cultivated as described above. For anthocyanin extraction, seedlings were germinated and grown for three days in a 0.5X MS liquid medium containing 1% sucrose under continuous light.

### Whole Genome Sequencing

MT and *atv*/*atv* (in MT background) genomic DNAs, extracted from single leaves using the “Wizard Genomic DNA Purification Kit” (Promega, Madison, WI, United States), were subjected to Whole Genome Sequence (WGS) analysis by IGATech (IGA Technology Services, Udine, Italy) using a HiSeq2500 System (Illumina, Inc., San Diego, CA, United States) with a 10x coverage. Bioinformatics analyses, including base calling and demultiplexing, alignment to the tomato Heinz 1706 Genome Reference (Tomato Genome Consortium, 2012), and variant calling [single nucleotide polymorphisms (SNPs), small InDels, structural variations] were performed.

### Phylogenetic Analysis

The analysis was performed on the Phylogeny.fr platform (Dereeper et al., 2008), by selecting the “One-click” mode. R3-MYB protein sequences were aligned with MUSCLE (v3.8.31) configured for highest accuracy (MUSCLE with default settings). Ambiguous regions (i.e., containing gaps and/or poorly aligned) were removed with Gblocks (v0.91b) using the following parameters: minimum length of a block after gap cleaning: 10; no gap positions were allowed in the final alignment; all segments with contiguous non-conserved positions bigger than 8 were rejected; minimum number of sequences for a flank position: 85%. The phylogenetic tree was reconstructed using the maximum likelihood method implemented in the PhyML program (v3.1/3.0 aLRT). The WAG substitution model was selected assuming an estimated proportion of invariant sites (of 0.145) and 4 gamma-distributed rate categories to account for rate heterogeneity across sites. The gamma shape parameter was estimated directly from the data (γ = 1.135). Reliability for internal branch was assessed using the aLRT test (SH-Like). The bootstrapping procedure is replaced by a different confidence index (Anisimova and Gascuel, 2006). Graphical representation and edition of the phylogenetic tree were performed with TreeDyn (v198.3).

### Cloning of the R3-MYB and bHLH Genes

The gene *Solyc07g052490* (*SlMYB-ATV*), encoding the R3-MYB protein SlMYB-ATV, was amplified by PCR starting from MT DNA using the “Phusion High-Fidelity DNA Polymerase” (Thermo Fisher Scientific, Waltham, MA, United States) and the oligonucleotide primers CACCATGGCAGATTGGAATAGATCAAGCACATCA and TTACTGGCTTTTGGAGTATTTTGATTTCC. The same gene was amplified using DNA extracted from *atv*/*atv* line in MT background (*Slmyb-atv*), LA0797 and LA3736 tomato lines and double mutant *Aft*/*Aft atv*/*atv*. The cDNA of *Slmyb-atv* was amplified by RT-PCR using the same primers described before from RNA extracted from leaves of *atv*/*atv* plants in AC background grown for two months under 12 h light photoperiod with a light intensity of approx. 300 μmol photons m^2^ s^-1^, and 24°C/21°C day/night temperature. The genes *Solyc09g065100* and *Solyc08g081140*, encoding the bHLH factors SlAN1 and SlJAF13 (Kiferle et al., 2015), respectively, were cloned starting from AC DNA using the same protocol above described and the primers CACCATGGAGATTATACAGCCTAATAG and TTAATTAACTCTAGGGATTATCTGATTTATTG (*SlAN1*) and CACCATGGCTATGGGACACCAAGATC and TCAAGATTTCCATACTACTCTCTGAAGTG (*SlJAF13*). The amplified sequences were cloned into pENTR/D-TOPO vector (Thermo Fisher Scientific) and the entry clones were recombined with different destination vectors, as described below, via Invitrogen^TM^ Gateway^TM^ recombination cloning technology (Thermo Fisher Scientific). The different amplified genes were sequenced for confirmation (Eurofins Genomics, Ebersberg, Germany). Multiple Sequence Alignments were performed using Multalin version 5.4.1 (Corpet, 1988).

### *Arabidopsis thaliana* and Tomato Plant Transformation

The 35S:*Solyc07g052490* construct was produced by recombining the *Solyc07g052490* entry clone with the Gateway^TM^ compatible binary vector pK7WG2^1^ (containing the cauliflower mosaic virus 35S promoter sequence) (Karimi et al., 2002). *Arabidopsis Col-0* plants ectopically expressing *Solyc07g052490* were produced via *Agrobacterium tumefaciens*-floral dip transformation method (Clough and Bent, 1998). Fourteen independent transgenic lines were analyzed in the T1 and T2 generations. Tomato transformation from cotyledons was carried out as described in Zuluaga et al. (2008). Three independent transgenic lines were produced in both wild type and *atv*/*atv* plants in MT background.

### Tomato Protoplasts Isolation

Tomato leaf protoplasts were isolated from 3-week-old MT plants following the protocol described in Shi et al. (2012). Polyethylene glycol-mediated protoplasts transformation was carried out as described in Yoo et al. (2007).

### Transactivation Assays

Transactivation assays by dual-luciferase system was performed exploiting the *Renilla reniformis* (Renilla) and *Photinus pyralis* (Firefly) luciferase (Luc) enzymes. The 35S:*SlANT1* and 35S:*SlAN2* effector constructs and the promoter *SlDFR*:*FireflyLuc* reporter construct were produced as reported in Kiferle et al. (2015). The 35S:*SlAN1* and 35S:*SlJAF13* effector constructs were produced by recombining the *SlAN1* and *SlJAF13* entry clones with the vector p2GW7 (Karimi et al., 2002). The promoter *SlMYB-ATV*:*FireflyLuc* reporter construct was produced after amplification of the 1,750 nucleotide (nt) sequence upstream of *Solyc07g052490* coding sequence (cds) with the primers CACCAAGAAAGGAACAAAAACAACAAA and TGAATTGGAGTACTAATTAAGGAAGAGTATATG, cloning in pENTR/D-TOPO vector and recombination with the reporter plasmid pPGWL7 (Kiferle et al., 2015). A 35S:*RenillaLuc* vector (Weits et al., 2014) was used to normalize luminescence values detected in protoplasts. Effector and reporter plasmids were co-transfected in tomato leaf protoplasts and luminescence relative levels were measured as described in Kiferle et al. (2015). In each experiment four biological replicates (independent protoplasts transformations) were used and each transactivation assay was repeated at least three times.

### Split-Luciferase Assay

The *SlMYB-ATV* and *Slmyb-atv* entry clones were individually recombined with the Gateway^TM^ compatible bait vector pDuEx-Ac6 (Fujikawa and Kato, 2007) containing the N-terminal half of the Renilla *luciferase* gene. The *SlAN1* and *SlJAF13* entry clones were individually recombined with the Gateway^TM^ compatible prey vector pDuEx-Dn6 (Fujikawa and Kato, 2007) containing the C-terminal half of the Renilla *luciferase* gene. Tomato leaf protoplasts were transfected with mixtures of two different recombined bait and prey vectors. As a control, the vector pDuEx-Ac6 in combination with each of the two recombined *SlAN1*- or *SlJAF13*-pDuEx-Dn6 vectors was used. Luciferase activity was analyzed using the ONE-Glo^TM^ Luciferase Assay System (Promega), following the manufacturer’s instructions. Luminescence was measured with a Lumat LB 9507 Tube Luminometer (Berthold Technologies GmbH & Co., KG, Bad Wildbad, Germany). Protein content of transfected protoplasts, measured by Bradford Protein Assay (Bio-Rad Laboratories, Hercules, CA, United States), was used for normalization of transfection efficiency. In each experiment four biological replicates (independent protoplasts transformations) were used for each combination of bait and prey vectors and each split-luciferase assay was repeated twice.

### Anthocyanin Quantification

Anthocyanin extraction was carried out as described in Kiferle et al. (2015). Relative anthocyanin concentrations were calculated as a difference between the absorbance read at 530 and 657 nm (Neff and Chory, 1998). The total amount of anthocyanins in tomato samples was expressed as mg petunidin-3-(p-coumaroyl-rutinoside)-5-glucoside g^-1^ fresh weight (FW), as described in Kiferle et al. (2015). Three biological replicates, consisting of single leaf samples or pooled leaves (as specified in the figure legends) from different plants, were used. In *Arabidopsis* seedlings anthocyanins were expressed as mg cyanidin-3-glucoside g^-1^ FW, based on an extinction coefficient of 29,600 and a molecular weight of 449 (Wrolstad et al., 2005). Three biological replicates, consisting of independent groups of seedlings, were used.

### Expression Analysis by Quantitative RT-PCR

Total RNA was extracted from *Arabidopsis* seedlings and tomato leaves using the “Spectrum Plant Total RNA Kit” (Sigma-Aldrich, St. Louis, MO, United States). RNA was subjected to DNase treatment and then reverse transcribed into cDNA using the “Maxima First Strand cDNA Synthesis Kit for RT-qPCR, with dsDNase” (Thermo Fisher Scientific). Quantitative RT-PCR (qPCR) was performed with an ABI Prism 7300 Sequence Detection System (Thermo Fisher Scientific) using the “PowerUp^TM^ SYBR^®^ Green Master Mix” (Thermo Fisher Scientific) and the primers listed in Kiferle et al. (2015) and Cao et al. (2017), as well as the primers AGGAAGGAAGCTACGATGATGCC and TGTCGGCTTTATCACTTCGTTCTC for *Arabidopsis SlDFR* gene (*At5g42800*) and the primers GATTGGAATAGATCAAGCACATCA and TTCGTTGGTAGTCTCTAATGCAAC for the *SlMYB-ATV* gene. *Arabidopsis Ubiquitin 10* (*At4g05320*) (Loreti et al., 2018), tomato *Elongation Factor 1-alpha* (*SlEF1A*) (Kiferle et al., 2015), and *Abscisic stress ripening gene1* (*SlASR1*) (Bovy et al., 2002) were used as reference genes. The relative quantification of gene expression was performed using the geometric averaging method (Vandesompele et al., 2002). In each experiment, three biological replicates (single mature leaf samples or pooled leaves from different plants, as specified in the figure legends) were used.

### Statistical Analysis

The GraphPad Prism Version 6.01 has been used to carry out the statistical analyses.

## Results

### Identification of a Candidate Mutant Gene in *atv*/*atv* Plants

**FIGURE 1:**
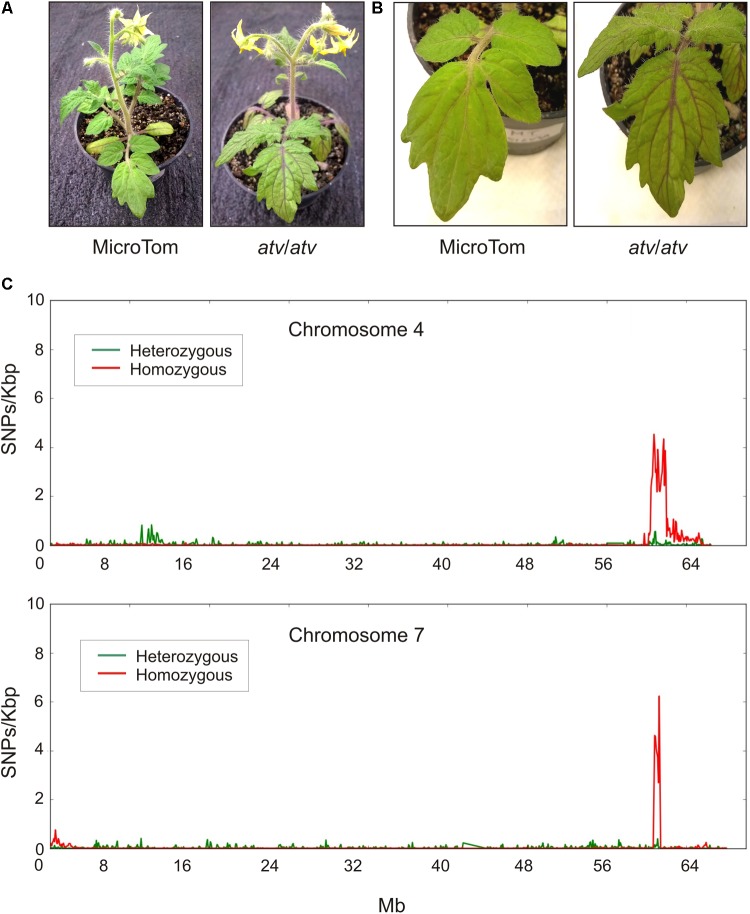
Tomato *atv* mutants show increased anthocyanins levels and contain extensive introgression regions in their genome. Phenotype of MicroTom (MT) and *atv*/*atv* plants (in MT background): **(A)** whole plants; **(B)** detail of leaves. **(C)** Distribution of the density of homozygous and heterozygous single nucleotide polymorphisms (SNPs) along chromosomes 4 and 7 of *atv*/*atv* genome.

The *atv* mutation, likely representing an allelic variant of a tomato gene coming from a wild *Solanum* species, was introgressed into different tomato genetic backgrounds. In MT, a near-isogenic line carrying a stable *atv* phenotype was produced (Sestari et al., 2014). *atv*/*atv* plants in MT background show increased anthocyanin accumulation in leaves and stems which is particularly evident in the leaf veins (**Figures 1A,B**). The genomic DNA of this *atv*/*atv* line was sequenced and aligned to the tomato reference genome (Tomato Genome Consortium, 2012). Many different polymorphic regions were detected scattered along all the chromosomes (Supplementary Figure 1), making impossible the identification of delimited mutated regions. The MT genomic DNA was thus sequenced too. All the polymorphisms found in both *atv*/*atv* and MT genomes were considered not involved in *atv* phenotype and thus filtered. In this way, most of the genomic variants identified through the first comparison with the reference disappeared (Supplementary Figure 2) and two delimited and specific polymorphic regions, whose lengths were approximately 6,071 Mb and 650 Kb, were finally identified, respectively, in chromosomes 4 and 7 of *atv*/*atv* (**Figure 1C**).

The presence of a putative introgressed segment in chromosome 7 confirmed previous segregation analyses carried out in the first described *atv*/*atv* mutant line (Rick et al., 1968; Clayberg, 1972). We therefore focused our analysis on this chromosome. Although short and well defined, this polymorphic region contained 71 annotated genes (Supplementary Table 1). Interestingly, most of them carried sequence variations in homozygosity (**Figure 1C**), suggesting a stable common inheritance. A candidate gene approach was thus carried out to identify genes showing high impact sequence structural variations and possibly connected with the anthocyanin pathway. Among the genes showing the highest impact variants (data not shown), a gene encoding a MYB protein, *Solyc07g052490*, was identified as our best candidate, being MYB TFs among the major regulators of anthocyanin synthesis. This gene in the *atv*/*atv* line showed a 4-nt insertion in the second exon as well as a SNP in the third exon, one in the 3^′^UTR and seven in the first intron (Supplementary Figure 3). While we were completing our experiments, a paper indicating the same gene as candidate for the *atv* mutation was published (Cao et al., 2017), strongly supporting our choice.

**FIGURE 2:**
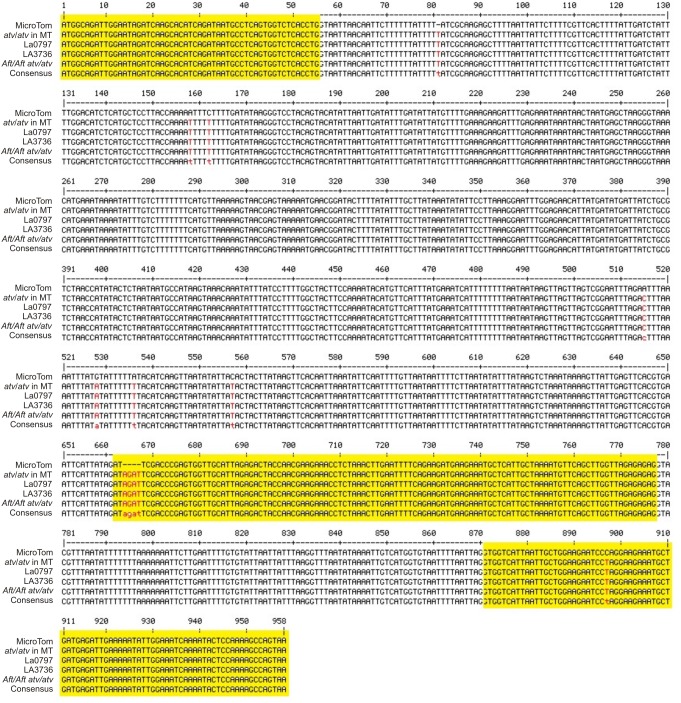
*atv* mutation in the gene *Solyc07g052490*. Multiple alignment of the gene *Solyc07g052490* (from start to stop codon) sequenced in different genetic backgrounds: MicroTom (MT), *atv*/*atv* in MT background, *atv*/*atv* in VF36 background (accession LA0797), *atv/atv* in Ailsa Craig (AC) background (accession LA3736), *Aft*/*Aft atv*/*atv*. Exons, as displayed in SOL Genomics Network database (https://sgn.cornell.edu/), are represented in yellow; SNPs and other sequence variants in red.

The gene *Solyc07g052490* was cloned and sequenced in the *atv*/*atv* line in MT background and in the two accessions LA0797 and LA3736 carrying the *atv* mutation in the VF36 and AC backgrounds. It was also sequenced in the double mutant *Aft*/*Aft atv*/*atv*. In all of these genotypes the gene showed the same polymorphisms already identified (**Figure 2**). As expected, the 4-nt insertion identified in the mutated *Solyc07g052490* gene was also found in the relative cDNA cloned in leaves from *atv*/*atv* plants (data not shown).

**FIGURE 3:**
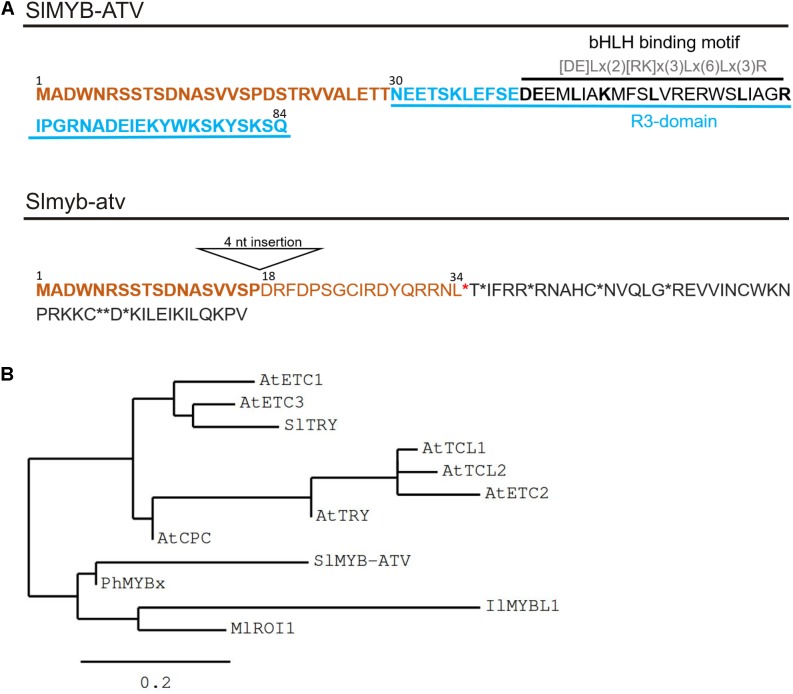
Main features of the SlMYB-ATV mutated protein. **(A)** Predicted protein sequences as result of the translation of the wild type (wt) allele (SlMYB-ATV) and of the *atv* allele (Slmyb-atv) of *Solyc07g052490*. N-terminal common aminoacids are marked in bold orange. In the wt version of the polypeptide, the R3 domain, containing the bHLH interaction motif, is indicated. In the *atv* version of the protein the position of the 18th amino acid, the first different from wt due to the 4 nucleotide (nt) insertion in the corresponding gene, is indicated. Stop codons are indicated by asterisks. **(B)** Phylogenetic tree showing the relatedness of SlMYB-ATV with other known plant R3-MYB proteins. The analysis was performed on the phylogeny.fr platform (Dereeper et al., 2008), using the maximum likelihood method (PhyML Program) and reliability for internal branch was assessed using the aLRT test (SH-Like). Graphical representation of the phylogenetic tree was performed with TreeDyn. Protein sequences were identified on the NCBI website and the relative GeneBank accession numbers are as follows: AtETC1 (OAP16560), AtETC2 (Q84RD1), AtETC3 (NP_192015), AtTCL1 (D3GKW6), AtTCL2 (B3H4X8), AtCPC (BAA21917), AtTRY (Q8GV05), SlTRY (MF197521), SlMYB-ATV (MF197509), PhMYBx (AHX24371), IlMYBL1 (ASR83103.1), MlROI1 (AGC66791.1).

*Solyc07g052490* encodes an 85 amino acid “MYB_related family protein” (PlantTFDB^2^; Jin et al., 2017), containing a single R3-MYB repeat domain. It was thus called *SlMYB-ATV* (Cao et al., 2017; **Figure 3A**). Interestingly, in the MYB domain, the SlMYB-ATV protein shows the conserved amino acidic signature [DE]Lx(2)[RK]x(3)Lx(6)Lx(3)R, through which MYB proteins can interact with bHLH factors (Zimmermann et al., 2004; **Figure 3A**).

The 4-nt insertion in the second exon of the *atv* version of *Solyc07g052490* (*Slmyb-atv*) leads to a frameshift and an early stop codon, with a strong impact in the corresponding polypeptide, which should be prematurely truncated, retaining only the wild type 19 amino acid N-terminal part (**Figure 3A**). It should also completely lack the R3-MYB domain including the above mentioned bHLH-interacting amino acidic domain (**Figure 3A**).

A phylogenetic study indicated the close relationship of SlMYB-ATV with other R3-MYB proteins involved in the negative regulation of anthocyanin synthesis in different plant species (Yuan et al., 2013; Albert et al., 2014a; Wang and Chen, 2014; Gates et al., 2017), particularly with PhMYBx of Petunia (**Figure 3B**).

### Correlation Between *SlMYB-ATV* Gene Expression and Anthocyanin Synthesis

**FIGURE 4:**
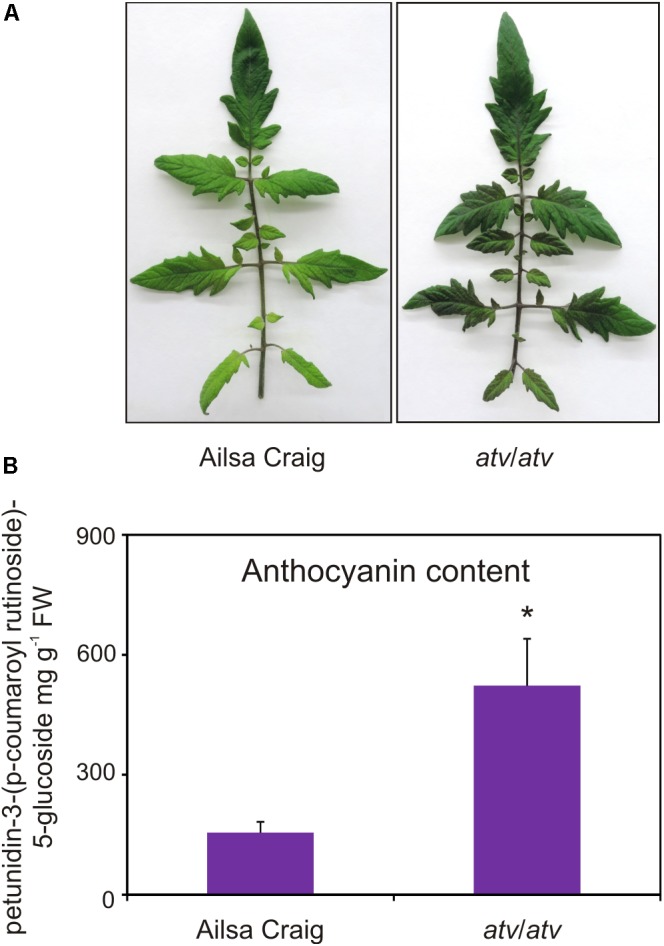
*atv* mutant plants grown under non-stressing conditions contain high basal levels of anthocyanins. **(A)** Phenotype and **(B)** anthocyanin content of leaves collected from Ailsa Craig (AC) and *atv*/*atv* mutant plants in AC background grown for two months under 12h light photoperiod with a light intensity of approx. 300 μmol photons m^2^ s^-1^, and 24°C/21°C day/night temperature. Anthocyanins are expressed in mg petunidin-3-(p-coumaroyl- rutinoside)-5-glucoside g^-1^ FW. Data are means of three biological replicates (single mature leaves from different plants) ± SE. Unpaired *t*-test (*P* < 0.05) was carried out and asterisks indicate significant differences.

**FIGURE 5:**
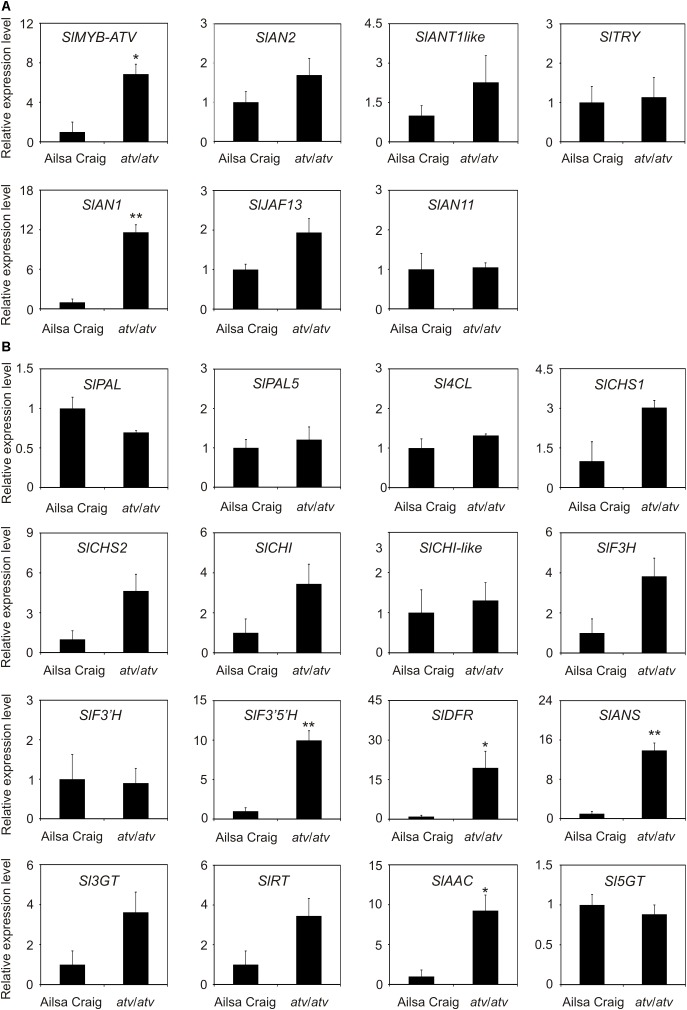
In *atv* mutant plants both regulatory and structural genes of the anthocyanin biosynthetic pathway are induced. Quantitative analysis of transcript levels of **(A)** regulatory and **(B)** structural genes in Ailsa Craig (AC) and *atv*/*atv* plants (in AC background) grown for two months under 12 h light photoperiod with a light intensity of approx. 300 μmol photons m^2^ s^-1,^ and 24°C/21°C day/night temperature. Expression levels, measured by qPCR, are shown as relative units, with the average value of the biological replicates of AC sample set to one. Data are means of three biological replicates (single mature leaves from different plants) ± SE. Unpaired *t*-test was carried out and single asterisks (*P* < 0.05) and double asterisks (*P* < 0.01) indicate significant differences.

AC and *atv*/*atv* plants in AC background accumulate very different amounts of anthocyanins (**Figure 4**), showing that, independently from the genetic context and being equal the external environment parameters, the *atv* mutation confers to tomato plants the ability of accumulating more anthocyanins. In the leaves of these plants we analyzed the transcripts of several genes involved in the anthocyanin biosynthesis (**Figure 5**). The genes participating in the regulation of the pathway, namely those encoding the R2R3-MYB activators SlAN2 and SlANT1like, the bHLH proteins SlJAF13 and SlAN1, and the WDR factor SlAN11(Kiferle et al., 2015; **Figure 5A**), as well as the structural genes, both EBGs and LBGs, encoding the enzymes of the pathway (**Figure 5B**), were found to be expressed. Also the gene *SlTRY*, encoding another tomato R3-MYB protein identified as regulator of anthocyanin synthesis (Tominaga-Wada et al., 2013) resulted expressed (**Figure 5A**). Only the gene encoding for the R2R3-MYB activator SlANT1 (Mathews et al., 2003) was below the level of detection, as previously observed in other tomato vegetative tissues (Kiferle et al., 2015). Coherently with the amount of anthocyanins measured in the leaves (**Figure 4B**), the anthocyanin biosynthetic pathway thus resulted more active in *atv*/*atv* plants, with many of the genes acting in the late steps (*SlF3^′^5^′^H*, *SlDFR, SlANS, SlAAC)* much more expressed in *atv*/*atv* than in AC. The same holds true for the bHLH activator *SlAN1* (**Figure 5A**). Remarkably, also the expression of the gene *SlMYB-ATV* resulted higher in *atv*/*atv* than in AC leaves (**Figure 5A**).

### Overexpression of *SlMYB-ATV* Reduces Anthocyanin Production

*Solyc07g052490* was cloned in MT plants and ectopically expressed under the control of a 35S promoter in *A. thaliana* and in tomato plants. In *Arabidopsis*, the ectopic expression of *SlMYB-ATV* induced a trichomeless phenotype and when anthocyanin synthesis was specifically induced by germinating and growing the seedlings under continuous light in a medium containing sucrose (Solfanelli et al., 2006), the amount of anthocyanins produced by the transgenic lines was lower than that produced by the wild type *Col-0* seedlings (Supplementary Figure 4). Furthermore, the expression of the LBG of the anthocyanin pathway *Dihydroflavonol 4-reductase* (*AtDFR*) was coherently downregulated (Supplementary Figure 4).

**FIGURE 6:**
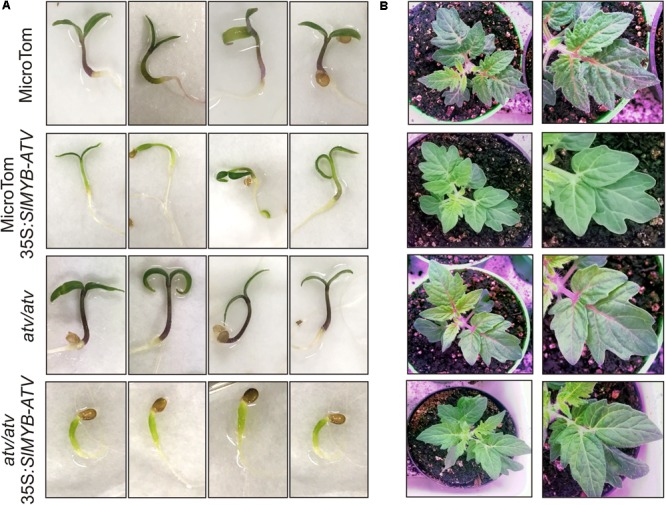
Overexpression of *SlMYB-ATV* in tomato. Phenotype of **(A)** 1-week-old seedlings and **(B)** 4-week-old plants of MicroTom (MT), 35S:*SlMYB-ATV* in MT, *atv*/*atv* in MT background and 35S:*SlMYB-ATV* in *atv*/*atv*.

**FIGURE 7:**
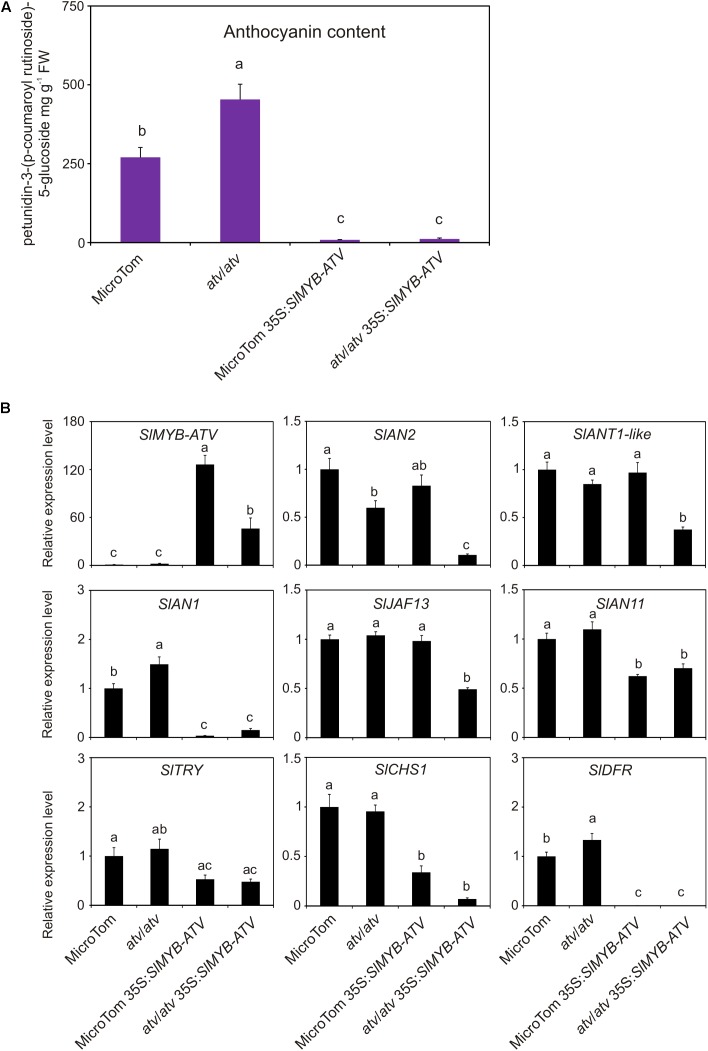
Overexpression of *SlMYB-ATV* in tomato plants abolishes anthocyanin accumulation. **(A)** anthocyanin content measured in leaves collected from 4-week-old MicroTom (MT), 35S:*SlMYB-ATV* in MT, *atv*/*atv* (in MT background) and 35S:*SlMYB-ATV* in *atv*/*atv* plants. Anthocyanins are expressed in mg petunidin-3-(p-coumaroyl-rutinoside)-5-glucoside g^-1^ fresh weight (FW). Data are means of three biological replicates (pooled leaf samples from three different 4-week-old plants/transgenic lines) ± SE. One-way ANOVA test (Tukey’s multiple comparisons test, *P* < 0.05) was carried out. Different letters indicate significant differences in each bar chart plot. **(B)** qPCR analysis of regulatory (*SlMYB-ATV*, *SlAN2*, *SlANT1-like*, *SlAN1*, *SlJAF13*, *SlAN11*, *SlTRY*) and representative structural genes (*SlCHS1*, *SlDFR*) of the anthocyanin biosynthetic pathway. Data are means of three biological replicates (pooled leaf samples from three different 4-week-old plants/transgenic lines) ± SE. One-way ANOVA test (Tukey’s multiple comparisons test, *P* < 0.05) was carried out. Different letters indicate significant differences in each bar chart plot.

In tomato, *SlMYB-ATV* was overexpressed in *atv*/*atv* and in WT plants in MT background (**Figure 6**). The overexpression of *SlMYB-ATV* led to a visible absence of anthocyanins since the seedling stage (**Figure 6A**). Moreover, transgenic lines in *atv*/*atv* background were delayed in the germination process and cotyledons encountered difficulties in exiting from the seed coat (**Figure 6A**). Once transplanted into soil, all transgenic lines grew as vigorously as the wild type plants, with no differences in terms of trichome density (**Figure 6B** and Supplementary Figure 5). The leaf anthocyanin content resulted strongly reduced in all the overexpressor lines, independently from the different content in the relative wild type genotypes (**Figure 7A**). A qPCR analysis confirmed the overexpression of the *SlMYB-ATV* gene in the transgenics (**Figure 7B**) and also indicated that the bHLH encoding gene *SlAN1* was strongly affected by SlMYB-ATV overexpression (**Figure 7B**). This also affected the R2R3-MYB genes *SlAN2* and *SlANT1-like*, as well as the bHLH *SlJAF13* genes, which resulted repressed in the overexpressor lines in *atv*/*atv* background, whereas the WDR-encoding gene *SlAN11* and the other R3-MYB gene *SlTRY* were found slightly repressed in all the transgenics (**Figure 7B**). Finally, both EBGs (exemplified by *SlCHS1*) and LBGs (represented by *SlDFR*) resulted strongly silenced when *SlMYB-ATV* was overexpressed (**Figure 7B**), coherently with the amount of anthocyanins measured (**Figure 7A**).

### SlMYB-ATV Inhibits the Activation of the Promoter of *SlDFR* and of Its Own Promoter

**FIGURE 8:**
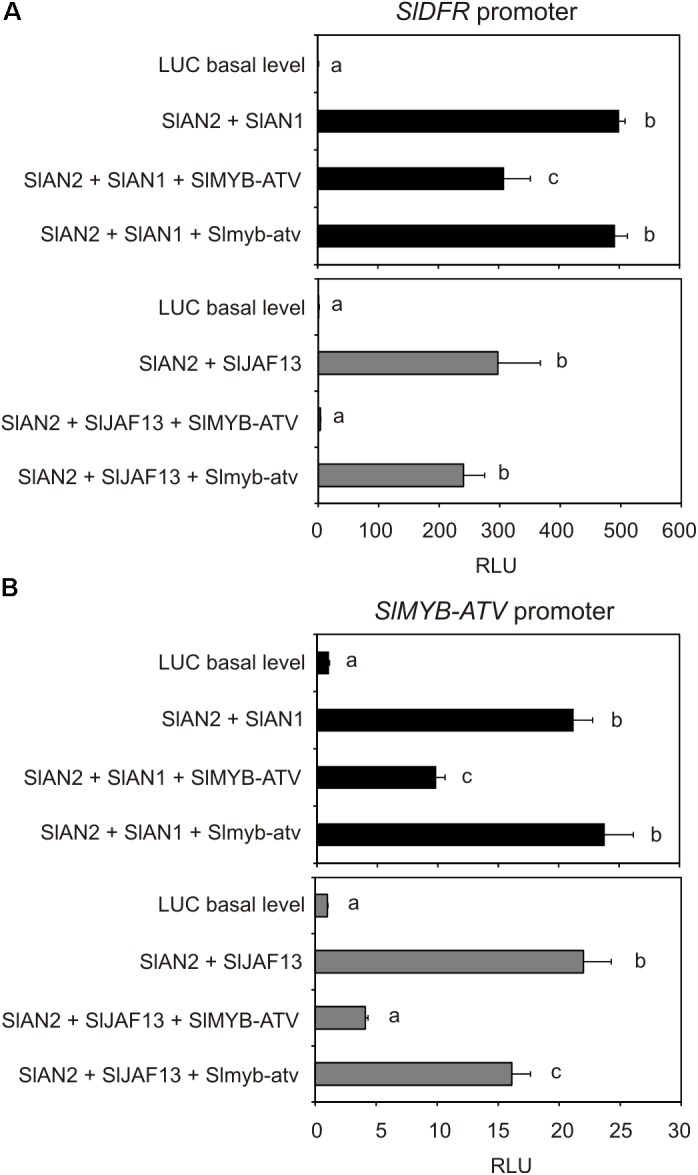
The transient expression of *SlMYB-ATV* represses both *SlDFR* and its own promoters. Dual luciferase assays in MicroTom leaf protoplasts transfected with the reporter plasmid containing **(A)** the *SlDFR* or **(B)** the *SlMYB-ATV* promoter driving *firefly luciferase* (*FireflyLuc*) gene alone (basal level histogram) or in combination with effector plasmids containing *SlAN2*, *SlAN1*, *SlJAF13*, *SlMYB-ATV*, and *Slmyb-atv* sequences, in different combinations. A 35S:*Renilla-luciferase* (*RenillaLuc*) plasmid was used as an internal control. Data are expressed as Relative Luciferase Activity (RLU) (FireflyLuc/RenillaLuc) and are means of four biological replicates ± SE. One-way ANOVA test (Tukey’s multiple comparisons test, *P* < 0.05) was carried out. Different letters indicate significant differences in each bar chart plot. Each experiment was repeated at least three times with similar results.

The transactivation of the promoter of the tomato *Dihydroflavonol 4-reductase* (*SlDFR*) gene by different effector constructs potentially able to reconstitute a MBW complex was analyzed. To form the complex, the protein SlAN2, a major R2R3-MYB-activator of tomato (Kiferle et al., 2015), in combination with one bHLH factor, SlAN1 or SlJAF13 (Kiferle et al., 2015), were transiently expressed. When each of these proteins was expressed individually, no activation of the reporter gene could be measured (data not shown). Only the two combinations SlAN2 + SlAN1 or SlAN2 + SlJAF13 significantly increased the luciferase activity, indicating that a MBW complex, able to bind the promoter of the structural gene *SlDFR* activating its transcription, had been actually formed, in combination with a WDR protein likely constitutively expressed in tomato protoplasts (**Figure 8A**). Remarkably, the concomitant overexpression of the SlMYB-ATV protein significantly reduced the activation of the *SlDFR* promoter by either of the two MBW complexes, in particular by the one containing the SlJAF13 bHLH factor (**Figure 8A**). Moreover, Slmyb-atv, the *atv* version of the R3-MYB protein produced by transient expression of the gene sequence cloned in *atv*/*atv* plants, was not able of performing a similar negative effect, indicating it was not functional (**Figure 8A**).

When a similar transactivation assay was carried out by using the promoter of the gene *SlMYB-ATV* to drive the expression of the reporter luciferase, a strong activation of the luminescence was obtained with either the MBW complexes containing SlAN2 + SlAN1 or SlAN2 + SlJAF13 (**Figure 8B**), showing that also the R3-MYB protein was under the transcriptional control by the two MBW complexes. At the same time, SlMYB-ATV could strongly inhibit the activation of its own transcription when expressed in combination with the two MBW complexes, showing again to be able to exert a negative interference with them (**Figure 8B**). Slmyb-atv mutated protein could only slightly affect the activation mediated by the MBW complexes of the promoter of the same *SlMYB-ATV* gene (**Figure 8B**).

### SlMYB-ATV Interacts With bHLH Tomato Factors Involved in MBW Complexes

**FIGURE 9:**
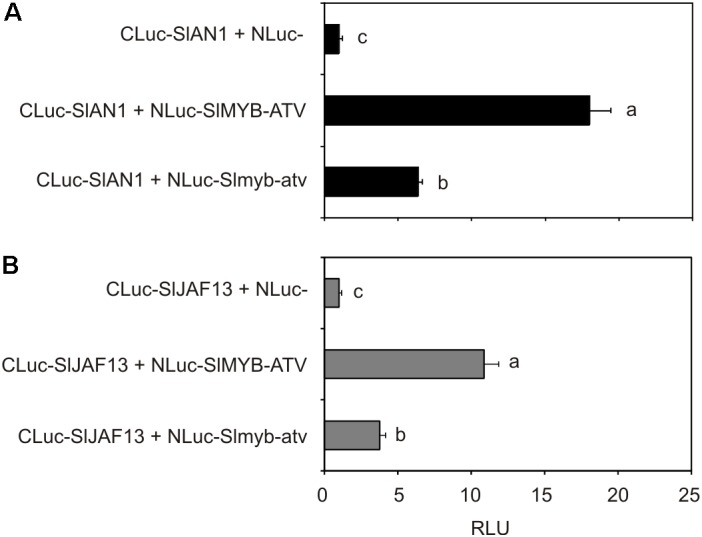
SlMYB-ATV can interact with tomato bHLH factors involved in anthocyanin synthesis. Split-luciferase assays in MicroTom leaf protoplasts expressing the fusion proteins NLuc-SlMYB-ATV or NLuc-Slmyb-atv with CLuc-SlAN1 **(A)** or CLuc-SlJAF13 **(B)**. As a control, NLuc-half protein was expressed in combination with each of the two CLuc-SlAN1 (A) or CLuc-SlJAF13 fusion proteins **(B)**. Data are expressed as Relative Luciferase Activity (RLU) (RenillaLuc/protein content) and are means of four biological replicates ± SE. One-way ANOVA test (Tukey’s multiple comparisons test, *P* < 0.05) was carried out. Different letters indicate significant differences in each bar chart plot. Each experiment was repeated twice with similar results.

To finally test the possible interaction between SlMYB-ATV and tomato bHLH factors participating in the MBW complexes regulating anthocyanin synthesis, tomato protoplasts were transfected with the *NLuc-SlMYB-ATV* fusion gene together with each of the two *CLuc-SlAN1* (**Figure 9A**) or *CLuc-SlJAF13* (**Figure 9B**) fusion genes. Luciferase activity was measured in both the cases (**Figure 9**), indicating that interaction between SlMYB-ATV and SlAN1 or SlJAF13 had occurred. When the Slmyb-atv mutated protein was expressed in place of SlMYB-ATV, a significant lower luciferase activity was measured, showing a weaker Slmyb-atv interaction compared to that of the wild type R3-MYB protein with either of the two bHLH factors (**Figures 9A,B**).

## Discussion

### *atv* Phenotype Is Associated With a Mutation in an R3-MYB Encoding Gene

The *atv* mutant of tomato is characterized by a strong anthocyanin pigmentation in stems, leaf veins and even green fruits, especially under cool conditions (Rick et al., 1968). Genome sequencing coupled with a candidate gene analysis approach allowed us to identify *Solyc07g052490* encoding a MYB protein, which is mutated in *atv*/*atv* plants (**Figures 1**–**3** and Supplementary Figures 1–3), as a candidate for the ATV identity. The mutation involves different sequence variants (**Figure 2** and Supplementary Figure 3), including one major insertion leading to a premature stop codon and thus to a possible polypeptide’s loss-of-function (**Figure 3**). Remarkably, the same gene was recently proposed as a candidate for ATV (named SlMYB-ATV, Cao et al., 2017).

MYB proteins belong to a very large family, mostly acting as TFs in plants. They are characterized by a conserved 50–53 amino acid “MYB” DNA-binding domain and a highly variable residual part (Li et al., 2016) and are classified into four major groups according to the number of MYB repeats (Dubos et al., 2010). The protein mutated in *atv*/*atv* plants belongs to a subgroup characterized by a single MYB domain (Du et al., 2013). The structure of the SlMYB-ATV protein and the presence of a conserved amino acidic signature through which MYBs can interact with bHLH factors (Zimmermann et al., 2004; **Figure 3A**) indicate that this protein belongs to the CPC-like subgroup, also called R3-MYB due to the homology of the MYB domain with the R3 repeat of the R2R3-MYB TFs (Du et al., 2013). R3-MYB proteins have been studied in many plant species where they are mainly involved in the regulation of cell fate determination (Wang and Chen, 2014), including down-regulation of anthocyanin synthesis (Koes et al., 2005; Zhu et al., 2009; Dubos et al., 2010; Albert et al., 2014a).

### Ectopic Expression of *SlMYB-ATV* Strongly Reduces Anthocyanin Production in Tomato

When ectopically expressed in *Arabidopsis* plants, *SlMYB-ATV* reduced the ability to accumulate anthocyanins by negatively regulating the expression of structural genes of the biosynthetic pathway (Supplementary Figure 4). Consistent with these results, the overexpression of *SlMYB-ATV* in tomato plants led to an anthocyaninless phenotype (**Figure 6** and Supplementary Figure 5). This resulted even more marked than the phenotypes exhibited by the *Arabidopsis* transgenic lines (Supplementary Figure 4), indicating that the negative interaction with the tomato endogenous anthocyanin regulatory mechanism could be more effective than that with its heterologous *Arabidopsis* counterparts.

Other plant R3-MYB proteins have been recently described as able to negatively regulate anthocyanin synthesis (Yuan et al., 2013; Albert et al., 2014a; Gates et al., 2017). In Petunia, PhMYBx is an R3-MYB protein which acts as a repressor able to sequester bHLH components from the MBW complex initiating anthocyanin biosynthesis (Albert et al., 2014a). Remarkably, a phylogenetic analysis carried out to compare SlMYB-ATV with other characterized plant R3-MYBs indicated a very close relationship with PhMYBx (**Figure 3B**; Cao et al., 2017).

The molecular phenotype shown by the tomato overexpressor lines, with a strong silencing of the gene encoding the regulatory bHLH factor SlAN1, whose direct effect was the downregulation of the anthocyanin biosynthetic genes (**Figure 7**), was opposite to that exhibited by *atv*/*atv* tomato plants, where, conversely, the high expression of the *SlAN1* gene was followed by the upregulation of the biosynthetic genes (**Figure 5**). In both the cases, an alteration of the mechanism activating the anthocyanin pathway, where MBW complexes play a primary role, was evident.

Excluding some delay in germination (**Figure 6**), the overexpression of *SlMYB-ATV* did not lead to other visible defect in tomato plants, whereas in *Arabidopsis* it clearly induced a trichomeless phenotype (Supplementary Figure 4). In *A. thaliana* seven genes encoding R3-MYB proteins have been described (Wang and Chen, 2014). They are mainly involved in epidermal cell patterning, regulating trichome and/or root hair development. The genetic control of these two processes in precise epidermal cells is similar and based on a multiprotein activator complex (made by specific R2R3-MYB, bHLH and WDR proteins), structurally similar to the ones involved in the regulation of anthocyanin synthesis (Ramsay and Glover, 2005). The glabrous phenotype of the *Arabidopsis* transgenic plants ectopically expressing *SlMYB-ATV* and the lower ability to synthesize anthocyanins are thus consistent with a possible inhibition of trichome formation and anthocyanin initiation by interference with the *Arabidopsis* MBW complexes initiating these two different cellular programs.

Tomato, differently from *Arabidopsis* which shows only one unicellular non-glandular type of trichomes, possess seven morphologically distinct trichome types, with non-glandular and glandular structures, whose genetic regulation is only partially known (Kang et al., 2016). In one recent study (Kang et al., 2014), loss-of-function of the flavonoid pathway enzyme chalcone isomerase (SlCHI1) in the *anthocyanin free* (*af*) mutant of tomato was associated with reduced density and activity of the type VI trichome glands, the most abundant in tomato leaves and stems, besides absence of anthocyanins and, unexpectedly, terpenoids. However, other data sustain the hypothesis that the genetic regulation of trichome (and root-hair) formation in *Arabidopsis* and other *Brassicaceae* is peculiar and therefore different from the regulatory networks acting in the other species, *Solanaceae* included (Serna and Martin, 2006). Confirming this, our results do indicate that the overexpression of the R3-MYB protein SlMYB-ATV does not interfere with the regulatory mechanisms of hair formation in tomato.

### SlMYB-ATV Inhibits the Production of Anthocyanins Mediated by MBW Complexes by Directly Binding the bHLH Factors

In tomato protoplasts SlMYB-ATV reduced the induction of the transcription of *SlDFR* (a key LBG of the anthocyanin pathway) mediated by endogenous MBW complexes (**Figure 8A**). This strongly supports a possible role of the R3-MYB protein as a competitive inhibitor of the MBW complexes (Zhu et al., 2009; Albert et al., 2014a). The *atv*-mutated version of SlMYB-ATV did not affect the activation of the *SlDFR* promoter (**Figure 8A**), indicating that the lack of the R3-MYB domain led to a loss-of-function of the protein. The results of the protein interaction assay carried out in tomato protoplasts demonstrated that SlMYB-ATV could actually bind both the endogenous bHLH factors SlAN1 and SlJAF13 with a significantly higher efficiency of its mutated version (**Figure 9**). The reduced capacity of Slmyb-atv to bind the bHLH factors may be due to the loss of the specific domain necessary to interact with them (**Figure 3A**).

Remarkably, the promoter of the gene *SlMYB-ATV* appeared to be under the transcriptional activation of the MBW complexes and at the same time under its own repression (**Figure 8B**), in a feedback mechanism probably aiming at reducing or inhibiting the production of anthocyanins once activated by an external input.

The putative model for the regulation of the anthocyanin synthesis in eudicots, as postulated by Albert et al. (2014a) based on the data obtained in Petunia, thus finds a full confirmation in our experimental results. SlMYB-ATV can indeed inhibit in a specific way the core MBW complex containing the proteins SlAN2, SlAN1, and the endogenous WDR factor (likely represented by SlAN11) (Kiferle et al., 2015), which directly activates the transcription of the structural genes of the biosynthetic pathway as well as the same *SlMYB-ATV* gene (**Figure 8**). This inhibition is likely due to the ability of SlMYB-ATV to directly bind SlAN1 (**Figure 9A**), thus subtracting this protein to the possibility to form new MBW complexes. We also found a strong inhibition exerted by SlMYB-ATV on the MBW complex composed by SlAN2 and SlJAF13 (SlJAF13 is equivalent to bHLH1 in the model of Albert et al., 2014a; **Figure 8**), which, in accordance to such model, should act upstream to induce the transcription of *SlAN1* (*SlAN1* is equivalent to *bHLH2*), in a reinforcement mechanism, and the formation of the core complex (Albert et al., 2014a). The direct binding of SlMYB-ATV with the bHLH factor SlJAF13 (**Figure 9B**), which is thus subtracted from its own MBW complex, is again likely the cause of the negative interference of SlMYB-ATV on the activity of the MBW complex itself.

The transcriptomic analysis carried out in 35S:*SlMYB-ATV* tomato lines (**Figure 7B**) confirmed the above described mechanisms. It indeed showed how the overexpression of the negative regulator of the pathway SlMYB-ATV, by negatively inhibiting both the MBW complexes, could lead to a very marked silencing of the *SlAN1* bHLH gene, mirrored by the almost complete repression of the key LBG *SlDFR* and consequent absence of anthocyanins (**Figure 7**).

The ectopic repression caused by the overexpression of SlMYB-ATV affected additional genes respect to those influenced in *atv*/*atv* plants (**Figures 5**, **7B**), such as the WDR *SlAN11* and the other R3-MYB *SlTRY*, indicating a more profound alteration of the feedback mechanisms regulating the anthocyanin level with possible consequences in other repression pathways. The overexpression of *SlMYB-ATV* in an *atv*/*atv* background also presented distinctive features compared to the overexpression in the wild type MT background, in the concomitant inhibition of the two R2R3-MYB activators *SlAN2* and *SlANT1-like* and of the bHLH *SlJAF13* (**Figure 7B**). Previous evidences about suppression of the transcription of R2R3-MYB TFs directly carried out by R3-MYB proteins, independent from those operating on the MBW complexes, were reported for some *Arabidopsis* R3-MYBs (Wang and Chen, 2014), even if the molecular mechanisms remain largely unknown.

**FIGURE 10:**
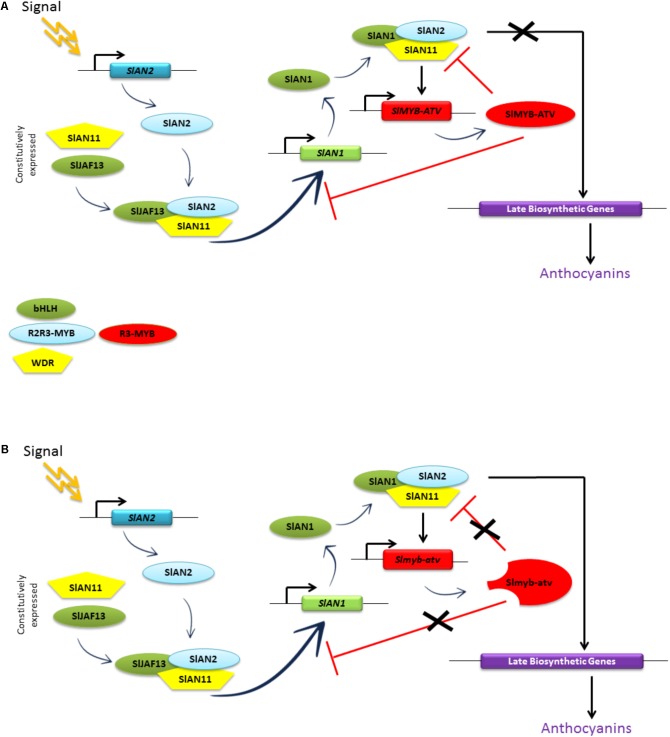
Model of the anthocyanin regulation in: **(A)** wild type and **(B)** *atv*/*atv* tomato plants. “Signal” indicates an inductive environmental or developmental stimulus that triggers anthocyanin production in tomato plants. Rectangles represent gene sequences; ovals and pentagons represent proteins. Adapted from Albert et al. (2014a).

**Figure 10A** summarizes the proposed mechanism of repression carried out by SlMYB-ATV on the MBW complexes of tomato. The higher level of anthocyanins produced by *atv/atv* plants (**Figure 4B**) can be explained by the reduced capacity of the mutated Slmyb-atv protein to negatively interfere with the MBW complexes initiating the anthocyanin synthesis (**Figure 10B**), due to the lower ability to directly bind in a competitive way the SlAN1 and SlJAF13 bHLH factors (**Figure 9**). The prolonged production of the pigments caused by the defect in the repressor protein which in the end cannot stop the reinforcement mechanism acting on the *SlAN1* transcription, can thus lead to a more sustained accumulation, attested by the higher expression level of all the genes which are under the transcriptional control of the MBW complexes (e.g., *SlAN1*, *SlDFR*, and the same *SlMYB-ATV* gene – **Figure 5**) and the higher amount of anthocyanins produced (**Figure 4**).

## Conclusion

In this paper we experimentally demonstrated that the *atv* phenotype of tomato mutants is genetically associated with a mutation of the gene *Solyc07g052490* encoding the R3-MYB protein recently named SlMYB-ATV (Cao et al., 2017). On the basis of the experimental results obtained, we proposed and demonstrated a mechanism by which this protein, acting as a competitive inhibitor of the R2R3-MYB activators of the MBW complexes, operates in a feedback loop involving the regulation of the activity of the MBW complexes themselves, finally controlling the amount of anthocyanins produced. The effects of the *atv* mutation, which in the end is an amplifier of the anthocyanin production, are visible wherever anthocyanin synthesis is triggered. In *atv* tomato lines these effects are limited to the vegetative parts of the plant, where the anthocyanin biosynthetic pathway is fully feasible. In those tomato lines, where thanks to other introgressed loci (such as *Aft* or *Abg*) the anthocyanin synthesis is active also in the fruit peel (Mes et al., 2008; Gonzali et al., 2009; Scott and Myers, 2012), the effects of the *atv* mutation can be much more striking, making possible the strong accumulation of the pigments also in the “purple” tomatoes produced by these plants.

Further work on the molecular interactions between SlMYB-ATV and each of the other positive regulators of the pathway will allow to define the exact hierarchy and stoichiometry of the different complexes operating in the system. Finally, the existence in the genome of the *atv* mutants of extensive introgression regions in both the chromosomes 4 and 7 proved by the present study (**Figure 1C**) requires additional analyses to understand if mutations in other loci can contribute to the phenotype of these tomato lines.

## Data Availability

The raw data supporting the conclusions of this manuscript will be made available by the authors, without undue reservation, to any qualified researcher.

## Author Contributions

SG and PP designed the experiments. SC performed the experiments. SG, SC, and PP analyzed data. SG wrote the manuscript.

## Conflict of Interest Statement

The authors declare that the research was conducted in the absence of any commercial or financial relationships that could be construed as a potential conflict of interest.

## Footnotes

1 http://gateway.psb.ugent.be/

2 https://omictools.com/plant-transcription-factor-database-tool

## Abbreviations

AC: Ailsa Craig
*Aft*: *Anthocyanin fruit*
*Atv*: *atroviolacea*
EBGs: early biosynthetic genes
LBGs: late biosynthetic genes
MBW: MYB-bHLH-WDR
MT: MicroTom
