# Supplementary figures and images for: The atroviolacea Gene Encodes an R3-MYB Protein Repressing Anthocyanin Synthesis in Tomato Plants
Source: Front Plant Sci. 2018 Jun 19;9:830. doi: 10.3389/fpls.2018.00830 (PMC6018089; doi:10.3389/fpls.2018.00830)

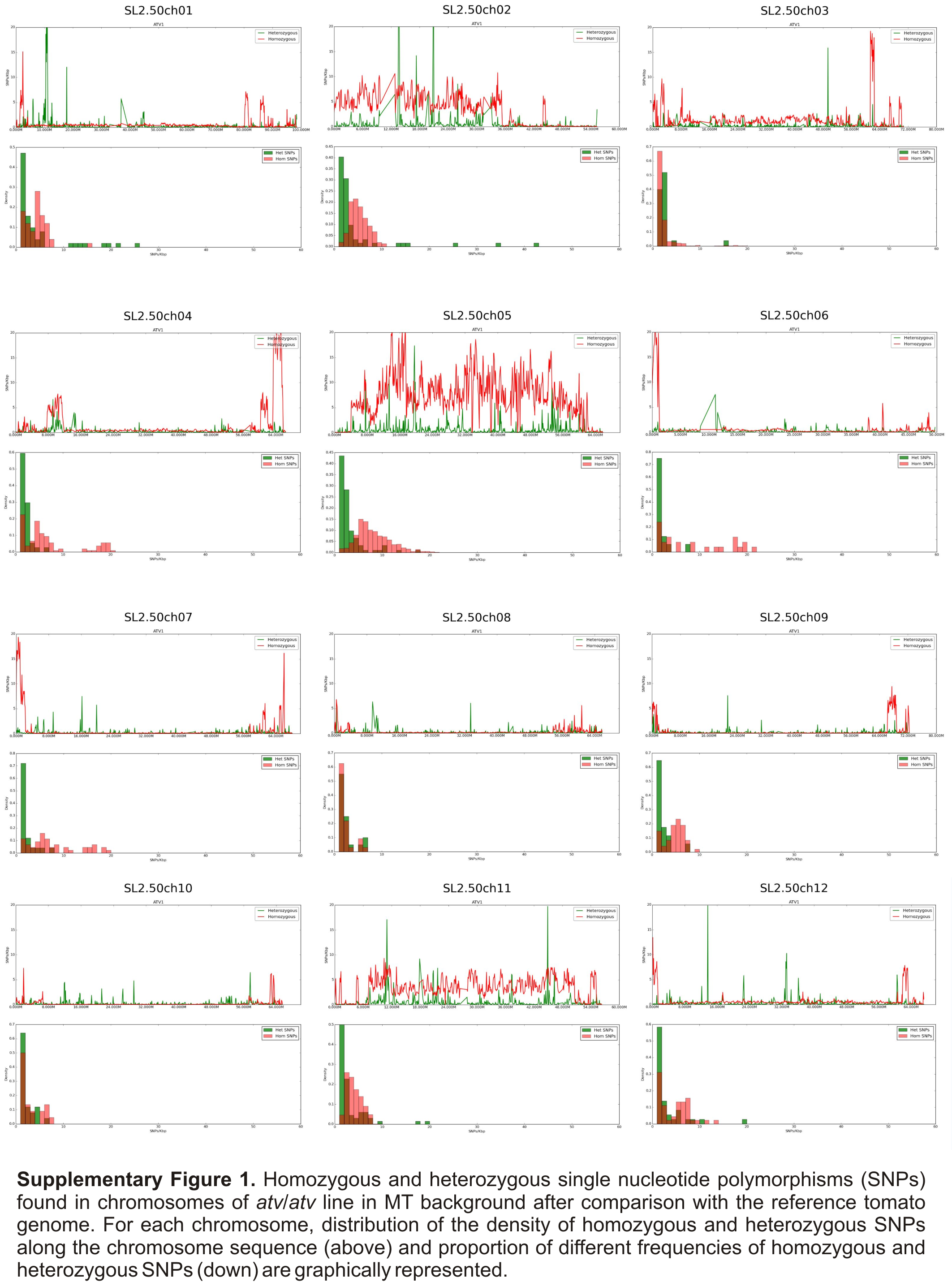

Supplement: Supplementary file 1 [file Image_1.TIF]

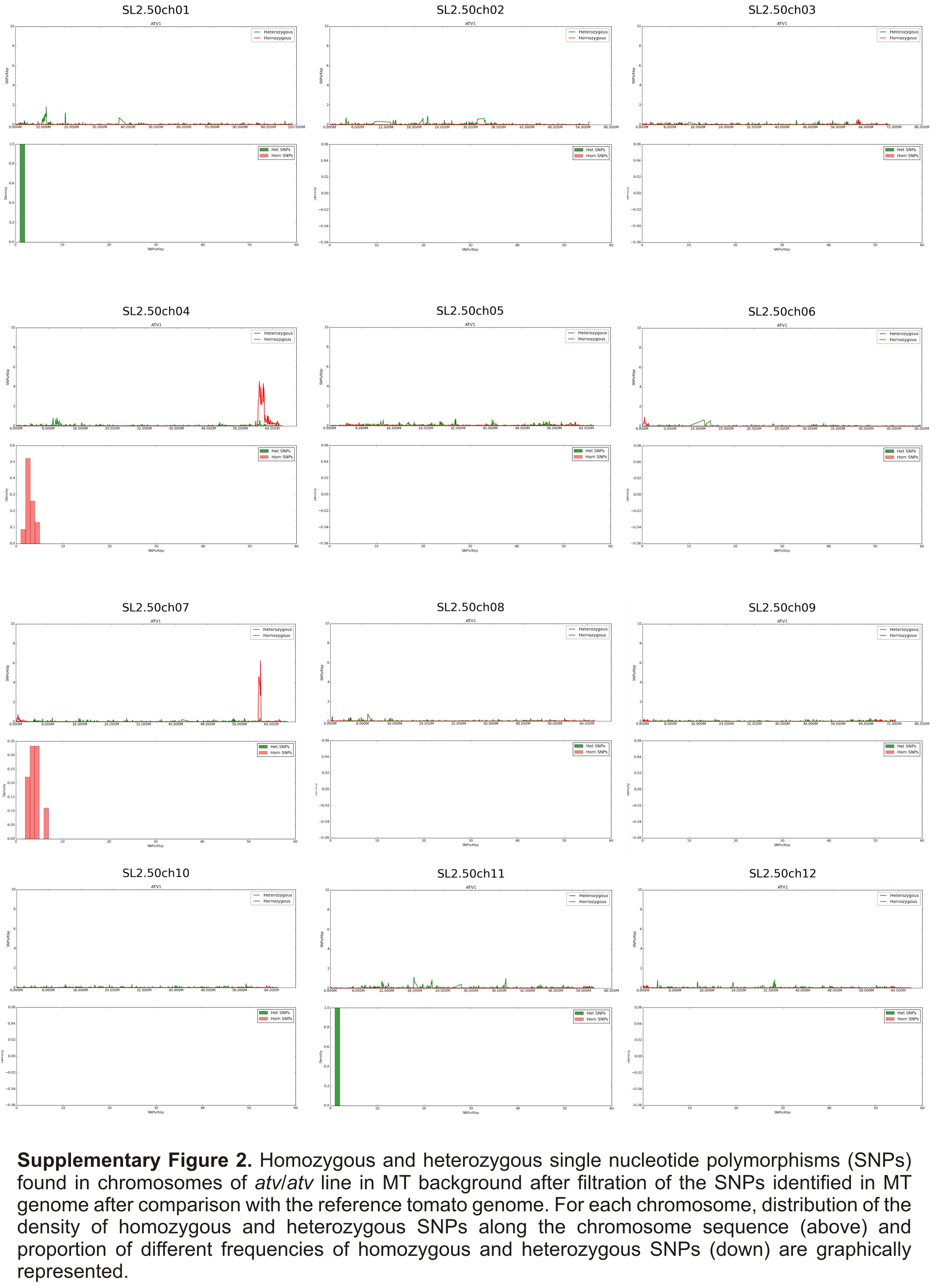

Supplement: Supplementary file 2 [file Image_2.TIF]

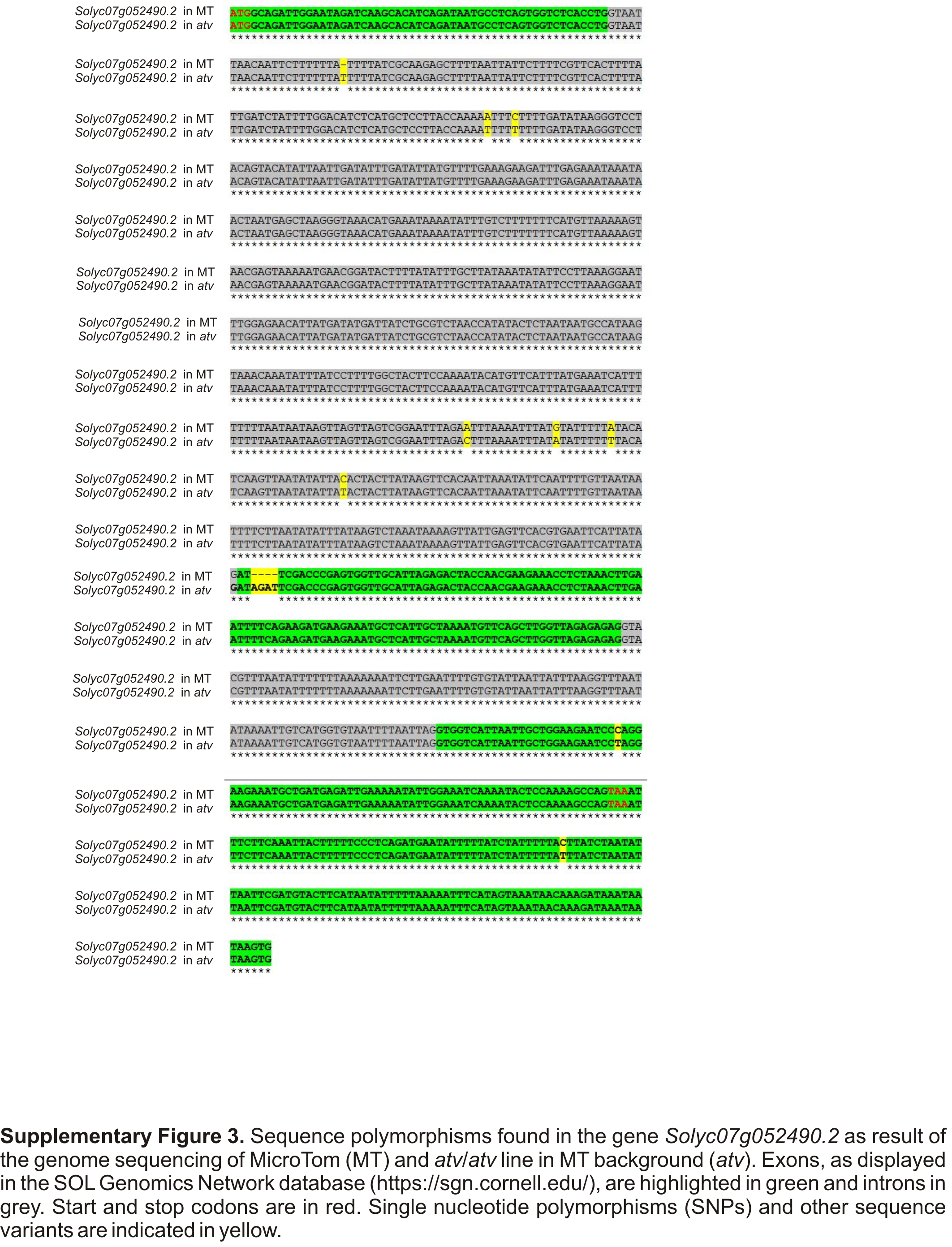

Supplement: Supplementary file 3 [file Image_3.TIF]

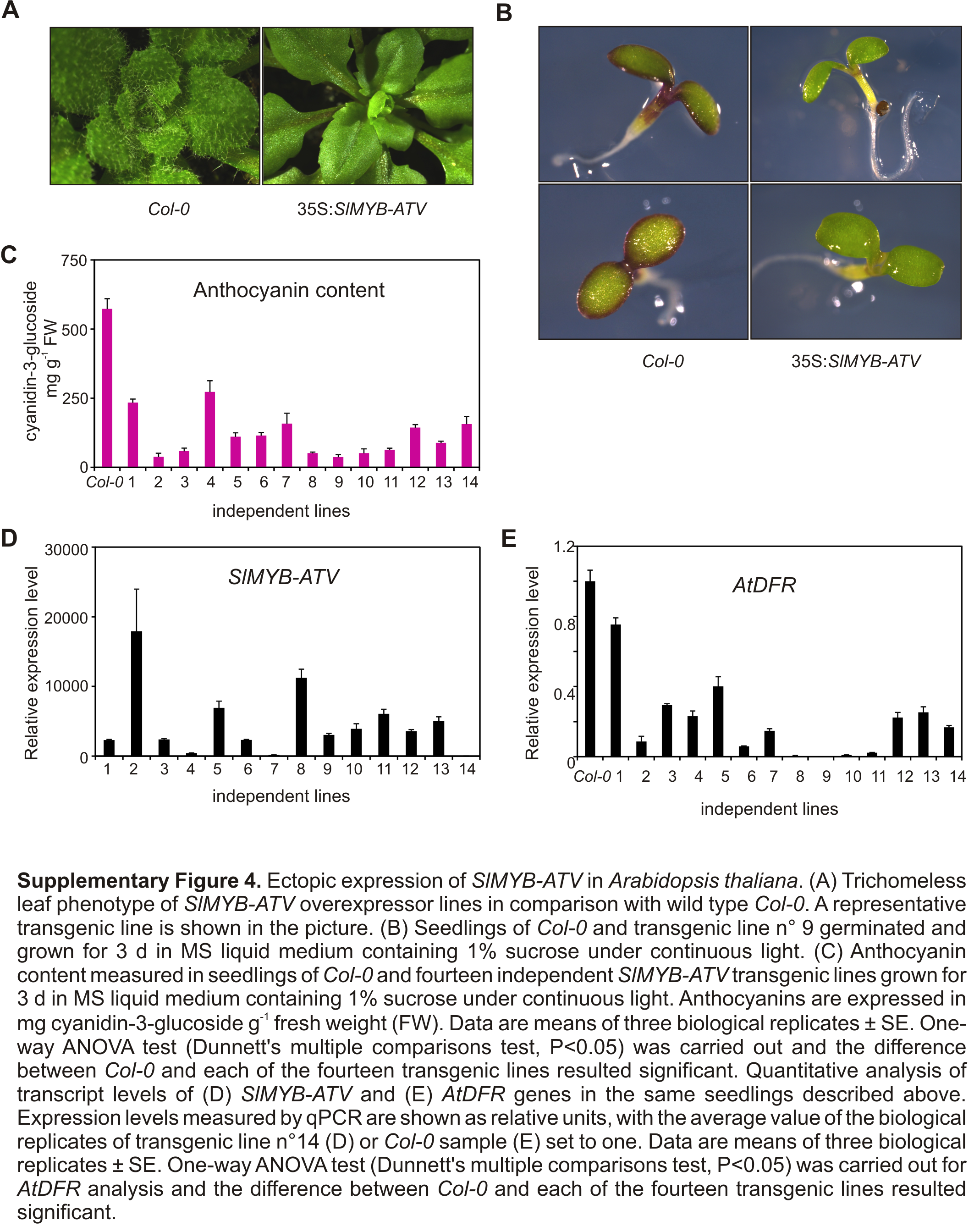

Supplement: Supplementary file 4 [file Image_4.TIF]

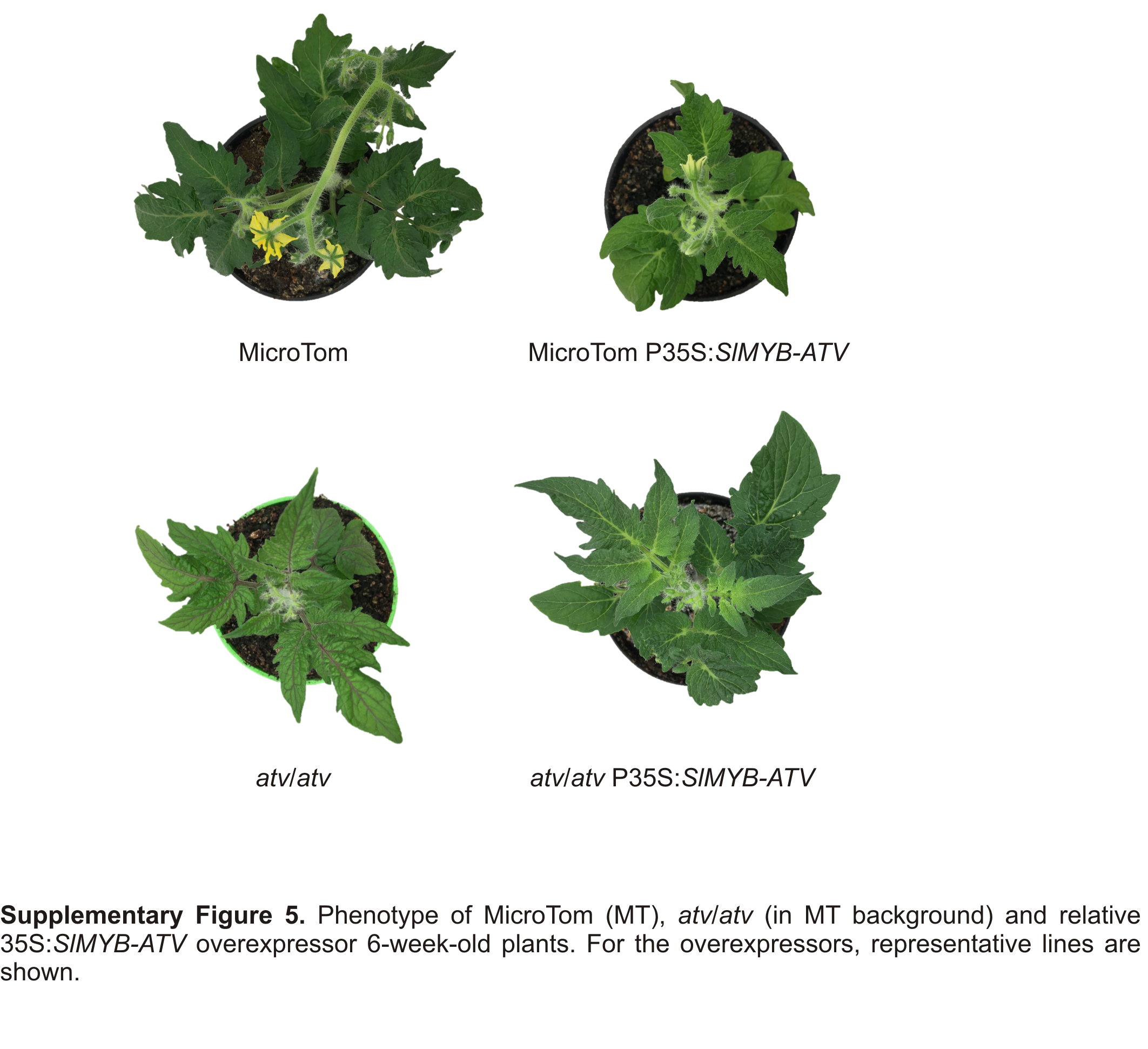

Supplement: Supplementary file 5 [file Image_5.TIF]
