# Supplementary material for: The atroviolacea Gene Encodes an R3-MYB Protein Repressing Anthocyanin Synthesis in Tomato Plants
Source: Front Plant Sci. 2018 Jun 19;9:830. doi: 10.3389/fpls.2018.00830 (PMC6018089; doi:10.3389/fpls.2018.00830)
Supplement: Supplementary file 7 [file Data_Sheet_1.DOCX]

Supplementary Material

The *atroviolacea* Gene Encodes an R3-MYB Protein Repressing Anthocyanin Synthesis in Tomato Plants

**Sara Colanero, Pierdomenico Perata*, Silvia Gonzali***

PlantLab, Institute of Life Sciences, Scuola Superiore Sant'Anna, San Giuliano Terme (Pisa), Italy

*** Correspondence:** P. Perata: p.perata@santannapisa.it; Silvia Gonzali: s.gonzali@santannapisa.it

**Supplementary data**

**Supplementary Table 1.** List of the genes mutated in chromosome 7 with relative protein functions

**Supplementary Figure 1.** Homozygous and heterozygous single nucleotide polymorphisms (SNPs) found in chromosomes of *atv*/*atv* line in MT background after comparison with the reference tomato genome. For each chromosome, distribution of the density of homozygous and heterozygous SNPs along the chromosome sequence (above) and proportion of different frequencies of homozygous and heterozygous SNPs (down) are graphically represented.

**Supplementary Figure 2.** Homozygous and heterozygous single nucleotide polymorphisms (SNPs) found in chromosomes of *atv*/*atv* line in MT background after filtration of the SNPs identified in MT genome after comparison with the reference tomato genome. For each chromosome, distribution of the density of homozygous and heterozygous SNPs along the chromosome sequence (above) and proportion of different frequencies of homozygous and heterozygous SNPs (down) are graphically represented.

**Supplementary Figure 3.** Sequence polymorphisms found in the gene *Solyc07g052490.2* as result of the genome sequencing of MicroTom (MT) and *atv*/*atv* line in MT background. Exons, as displayed in the SOL Genomics Network database (https://sgn.cornell.edu/), are highlighted in green and introns in grey. Start and stop codons are in red. Single nucleotide polymorphisms (SNPs) and other sequence variants are indicated in yellow.

**Supplementary Figure 4.** Ectopic expression of SlMYB-ATV in *Arabidopsis thaliana*. (A) Trichomeless leaf phenotype of SlMYB-ATV overexpressor lines in comparison with wild type *Col-0*. A representative transgenic line is shown in the picture. (B) Seedlings of *Col-0* and transgenic line n° 9 germinated and grown for 3 d in MS liquid medium containing 1% sucrose under continuous light. (C) Anthocyanin content measured in seedlings of *Col-0* and fourteen independent *SlMYB-ATV* transgenic lines grown for 3 d in MS liquid medium containing 1% sucrose under continuous light. Anthocyanins are expressed in mg cyanidin-3-glucoside g-1 fresh weight (FW). Data are means of three biological replicates ± SE. One-way ANOVA test (Dunnett's multiple comparisons test, P<0.05) was carried out and the difference between *Col-0* and each of the fourteen transgenic lines resulted significant. Quantitative analysis of transcript levels of (D) *SlMYB-ATV* and (E) *AtDFR* genes in the same seedlings described above. Expression levels measured by qPCR are shown as relative units, with the average value of the biological replicates of transgenic line n°14 (D) or *Col-0* sample (E) set to one. Data are means of three biological replicates ± SE. One-way ANOVA test (Dunnett's multiple comparisons test, P<0.05) was carried out for *AtDFR* analysis and the difference between *Col-0* and each of the fourteen transgenic lines resulted significant.

**Supplementary Figure 5.** Phenotype of MicroTom (MT), *atv*/*atv* (in MT background) and relative P35S:*SlMYB-ATV* overexpressor 6-week-old plants. For the overexpressors, representative lines are shown.
